# Strategies Implemented by 20 Local Tobacco Control Agencies to Promote Smoke-Free Recreation Areas, California, 2004-2007

**Published:** 2011-08-15

**Authors:** Travis D. Satterlund, Diana Cassady, Jeanette Treiber, Cathy Lemp

**Affiliations:** Center for Evaluation and Research, University of California, Davis; Center for Evaluation and Research, University of California, Davis, Davis, California; Center for Evaluation and Research, University of California, Davis, Davis, California; Center for Evaluation and Research, University of California, Davis, Davis, California

## Abstract

**Introduction:**

Since 2000, local jurisdictions in California have enacted hundreds of policies and ordinances in an effort to protect their citizens from the harmful effects of secondhand smoke. We evaluated strategies used by state-funded local tobacco control programs to enact local smoke-free policies involving outdoor recreational spaces.

**Methods:**

The Tobacco Control Evaluation Center analyzed 23 final evaluation reports that discussed adopting local smoke-free policies in outdoor recreational facilities in California. These reports were submitted for the 2004 through 2007 funding period by local tobacco control organizations to the California Department of Public Health, Tobacco Control Program. We used a comparative technique whereby we coded passages and compared them by locale and case, focusing on strategies that led to the enactment of smoke-free policies.

**Results:**

Our analysis found the following 6 strategies to be the most effective: 1) having a "champion" who helps to carry an objective forward, 2) tapping into a pool of potential youth volunteers, 3) collecting and using local data as a persuasive tool, 4) educating the community in smoke-free policy efforts, 5) working strategically in the local political climate, and 6) framing the policy appropriately.

**Conclusion:**

These strategies proved effective regardless of whether policies were voluntary, administrative, or legislative. Successful policy enactment required a strong foundation of agency funding and an experienced and committed staff. These results should be relevant to other tobacco control organizations that are attempting to secure local smoke-free policy.

## Introduction

In the past 2 decades, a growing body of scientific evidence has demonstrated the dangers of secondhand smoke (1,2). New evidence showing that secondhand smoke in outdoor areas also presents health risks (3) has elevated efforts to control tobacco use in outdoor spaces and recreational areas to protect people from exposure to secondhand smoke. Furthermore, prohibiting smoking in parks, on beaches, and in other outdoor recreation areas reduces tobacco litter (ie, cigarette butts) and its resulting toxins, which can be harmful (4), and discourages adults from modeling smoking, which may influence youth (5). Finally, most US citizens support smoke-free policy in many outdoor recreation areas (6,7). For all these reasons, securing outdoor smoke-free policy has become a recommended strategy by the Centers for Disease Control and Prevention (CDC) for states and local jurisdictions (8), and California's tobacco control program likewise supports community policies to restrict outdoor area smoking (9).

Policy theorists and tobacco control researchers provide some guidance in their analysis of factors that lead to successful local policy change (10-12). Many variables may explain adoption of policy, including the support of key decision makers, emulation of comparable jurisdictions (13), and alignment of the policy with the local political climate (12,14). Affluent communities appear more likely to support costly local policies (12-14). There is also evidence that the presence of "political entrepreneurs" and strong advocacy organizations facilitate policy adoption (15,16). Finally, the likelihood of local policy adoption of smoking-related laws is higher when the local health community promotes the efforts (17).

Since 2000, local jurisdictions in California have enacted hundreds of policies and ordinances in an effort to protect their citizens from the harmful effects of secondhand smoke. The California Tobacco Control Program (CTCP) funds tobacco control programs of county health departments statewide, a small number of metropolitan areas, and selected community-based organizations, and supports the Tobacco Control Evaluation Center (TCEC) of the University of California, Davis. CTCP coordinates a comprehensive approach of multifaceted strategies; its goal is "to change the broad social norms around the use of tobacco," and by doing so "creating a social environment and legal climate in which tobacco use becomes less desirable, less acceptable, and less accessible" (18). Local projects focus on 1 or more programmatic objectives, such as implementing tobacco retail licensing, limited smoking in apartment complexes, and increasing the number of smoke-free outdoor spaces and recreational facilities. At the conclusion of their work, they are required by CTCP to submit final evaluation reports (FERs), which TCEC analyzes and scores.

Despite the evidence of the many external factors that are related to adoption of tobacco control policies, little is known about the strategies local organizations use to change local policies. Our objective was to analyze local policy initiatives to identify common strategies used to adopt smoking policies in outdoor spaces and recreational areas at the local level in California.

## Methods

FERs followed a standard format that included an abstract, introduction, intervention activities, evaluation methods and results, and conclusions and recommendations. Each main section included subsections of required information. TCEC scored FERs to ensure accountability and to offer feedback to the local projects for future tobacco control evaluations. Of the 400 FERs submitted, we selected only those with an objective related to adopting smoke-free policy in outdoor spaces and recreational areas for analysis. Outdoor spaces and recreational areas included parks, beaches, piers, sport stadiums, and places where other local community events are held, including fairs, rodeos, and parades. Local projects submitted FERs in June and July of 2007, and we analyzed them between May and September of 2010.

We used a qualitative data analysis with an evolving coding scheme (19) to identify strategies employed in promoting smoking policies in outdoor spaces and recreational areas. Local FERs were analyzed, and variables emerged during the coding process. The data used for this study were collected from 23 FERs that 20 local tobacco control projects submitted to CTCP for the 2004 through 2007 funding period.

After identifying the relevant FERs, a reviewer used open coding, whereby "similar events/actions/interactions are grouped together to form categories and subcategories" (20). As in grounded theory field work (21), categories emerged from the reading of the first papers through "questioning and constant comparison" and were modified and expanded with the reading of subsequent papers. This quasi-inductive, pattern-level analysis considered the frequency, weight, and contextual factors of items across FER data (22). The reviewer performed several readings of all reports. After the reviewer had coded all reports, she created summary tables and narratives. Then, a second reviewer read all reports to confirm or dismiss any categorization, to look for trends that were missed, and to seek evidence that conflicted with the first reviewer's findings (23). Two other members of the research team then compared the results to findings in existing literature.

## Results

Nineteen of the 20 projects passed some form of policy to promote smoke-free recreational facilities (Table). The 1 project unable to secure passage of an official policy did succeed in having its county board of supervisors approve a preliminary plan. Some projects advocated for voluntary or administrative changes that could be made without a formal vote, such as making a music festival or a ski resort smoke-free. Other projects sought policy changes with local fair boards to add smoke-free days to county fairs or rodeos, which typically required a formal vote of the respective boards. Policies pursued by projects working with city councils ranged from seeking a single smoke-free park to enacting smoke-free policies that affected all parks and recreational areas in the city's jurisdiction. In 2 cases, policies legislated at the county level provided the impetus to municipalities in the county to create and enact their own citywide ordinances. Projects unable to secure stringent legislative policies sometimes decided to pursue more "doable" administrative or voluntary policies in their place.

Securing passage of policy was often difficult. The FERs described various challenges, including polarization related to perceived economic and individual rights issues, difficulties in gaining access to policy makers, internal organizational barriers such as staff turnover, and local political orientation that centered around conservative versus liberal viewpoints of the proposed policies (24).

Six strategies for success emerged from our analysis: 1) having a "champion" who helped to carry an objective forward, 2) tapping into a pool of potential youth volunteers, 3) collecting and using local data as a persuasive tool, 4) educating the community in smoke-free policy efforts, 5) working strategically in the local political climate, and 6) framing the policy appropriately.

### Having a champion

A crucial factor in a project's success was identifying a "champion," someone who was a member of, or was respected by, the targeted decision makers. FERs repeatedly mentioned examples of champions helping to carry a policy forward. An FER from a coastal county noted, "For us, the most crucial component of a successful smoke-free beach/pier policy effort has proven to be a strong champion." Champions typically had inside knowledge of the decision-making body and had the power to influence the outcome of policy adoption or provided access to policy makers, a critical step in adopting policy (25). A rural county wrote, "The fair director was crucial for us. He . . . supported the concept of a smoke-free fair and was key to developing the good working relationships with the fair."

### Tapping potential youth volunteers

Projects recruited youth volunteers from local universities, colleges, and high school groups already engaged in antidrug work. These students lent their enthusiastic presence to all phases of the campaign: planning, collecting data, and making presentations to the policy makers. Youth were often seen as the primary benefactors of such laws in places such as parks, beaches, and stadiums and for events such as fairs and carnivals, so successful projects often framed their policy arguments around the safety of children. Having the youth make these presentations was a strategic coup. One county's FER stated

Project staff showed great political acumen in collecting data and having youth present the data to the city councils. They operated on the assumption that it would be difficult for public officials and role models to refuse to act in what was demonstrated to be clearly in the best interest of both youth and the public at large.

### Using local data as a persuasive tool

Before meeting with policy makers, most local agencies gathered national and state fact sheets and polling results from tobacco-related networks. This information was augmented with local data such as public opinion surveys to demonstrate community support and key informant interview data that provided insights into policy makers' positions on the same issues. FERs made it clear that local decision makers found data gathered from the community itself persuasive. For example, the effect on policy makers of 30 student volunteers gathering more than 22,000 cigarette butts in 2 hours was profound and dramatically underscored the need for smoke-free policy. This and other forms of local data also helped establish a baseline for later comparisons.

### Educating the community

Community education was essential in securing support for local outdoor smoke-free policies. Local projects set up information booths at public events and made presentations to community organizations, vividly describing the harmful effects of tobacco litter and secondhand smoke.

Many projects took full advantage of local media (ie, newspapers, radio, and television) by using inventive approaches to bring the need for tobacco control directly to the community and simultaneously building local support and placing pressure on decision makers. One report from a rural county described this strategy as follows:

The strategic timing of ads, articles, letters to the editor, and op-eds helped to propel smoke-free parks onto the community's and elected officials' radar leading up to critical policy discussions and votes.

### Working strategically in the local political climate

Each community faced unique issues related to local policy makers and the political climate. Key informant interviews with policy makers, influential constituents, and other community insiders were crucial in developing and maintaining an effective campaign, as one rural county's FER explained:

Key informant interviews at the prepolicy adoption phase provided us with some of the challenges facing our adoption of smoke-free outdoor area policies. Respondents also gave us . . . their opinion on what strategies might work to counter these challenges. For instance, after conducting these interviews we were able to obtain specific information about some of the city officials who we would need to focus our efforts on because they were seen as opposing smoke-free policies due to special vested interests.

Several FERs noted that constituencies in the same county aligned along different, often opposing, viewpoints, requiring carefully adapted approaches to accomplish the desired results. Recognizing and respecting the political climate that shaped the thinking of each decision maker, rather than attempting to use a generic, one-size-fits-all strategy, was necessary. Several FERs described recasting the argument for smoke-free parks and beaches to frame the issue less around the dangers of secondhand smoke and more around environmental issues caused by cigarette litter. Particularly in coastal regions and among more affluent populations, the environment "held more weight," as one FER put it. Successful strategic shifts often resulted from a deeper understanding of the local political climate.

### Framing the policy appropriately

Three tactics emerged as particularly successful framing devices in persuading decision makers to support the proposed policy: 1) demonstrating constituent support for the proposed policy, 2) conveying how the proposed policy protected children and the environment, and 3) demonstrating that precedent existed. The most successful presentations that the FERs described tended to incorporate these 3 ideas.

Presentations, generally led by project directors or coordinators, frequently involved the active support of coalition members and community volunteers bolstered by the legitimizing presence of representatives from local chapters of national organizations such as the American Cancer Society and the American Lung Association. A strong emotional appeal for the protection of ecological values and young children from tobacco litter, backed by consistent data showing that the community favored tobacco control, frequently succeeded in eliminating opposition to the proposed policy. One county FER reported

Public presentations afforded us the ability to publicly deliver our data regarding exposure to secondhand smoke in outdoor areas, and our coalition members who helped us were able to gain a measure of public accountability from the officials and staff to address the issue.

Local projects also found that decision makers were swayed by legitimizing precedents, such as evidence of public acceptance for the same policy in neighboring communities or past tobacco-related successes in their own jurisdiction. One project director from a county with an urban populace wrote in the county's FER

In every case, mention was made of other communities that had implemented similar ordinances and measures. This effect cannot be overstated: no local action would have been likely without the evidence of reasonableness afforded by precedent. The presence of pending state-level legislation provided a similar legitimacy to local control efforts.

## Discussion

For the most part, our findings confirmed the results from other studies on policy change. For instance, several communities benefited from being near cities or counties that had already passed outdoor smoking bans, normalizing the issue for policy makers (26). The policy literature concludes that less affluent rural areas may face resistance to tobacco control regulations (12), which may explain why projects located in rural, less affluent counties were more likely to pursue weaker voluntary and administrative policy changes. Our findings that local health departments tailored policies to the local political climate and that they enlisted an influential champion to take up the cause follow the conclusions of existing literature (12,14-16), as does the influence of a health agency in the advocacy process (17). Also consistent with findings from other studies on tobacco control policy at the local level (14,26), we found no reports of tobacco industry lobbying.

This study identified 2 effective strategies used in local policy change not previously mentioned in other studies: 1) using local data to advance a policy position to decision makers, particularly displaying cigarette litter as a visual representation of the environmental problems that smoking causes, and 2) training youth advocates to collect cigarette litter and to present their findings to policy makers. These strategies are relevant for environmental policy change, which tended to appeal to constituents and decision makers across the political spectrum. In essence, these strategies enabled local projects to frame the issue around the more intuitively compelling issue of environmental protection as opposed to the less persuasive issue of secondhand smoke, which is perceived by the public and by policy makers as an indoor, not an outdoor, health problem. In many cases, policy makers who opposed the latter point on the basis of economic or individual-rights arguments (24) could still agree with a proposed outdoor smoke-free policy in the context of more wide-ranging local environmental concerns. Likewise, using youth volunteers to present the policy argument to decision makers tended to fortify the value of these policy positions.

This study also offers a more nuanced view of local tobacco control policy change, where policies are enacted not only by city councils or county boards of supervisors, but also by rodeo and fair boards, organizers of outdoor fairs and concerts, and administrators of parks and recreation districts. Policies can be legislative changes passed by elected officials, voluntary changes by concert organizers, or administrative changes by a host of different local agencies and organizations. The range of voluntary to legislative policies is in keeping with the "continuum of tobacco control policy" concept of Francis et al that progresses over time from voluntary to enforceable legislative policy (9).

One unexpected finding was the similarity of many of the policy strategies used in outdoor spaces and recreation areas to policy strategies used in other tobacco control policy campaigns. Blaine et al reached similar conclusions from their evaluation of 7 communities that successfully used similar methods to reduce youth access to tobacco. The authors concluded that a general set of strategies could be applied successfully to almost any "narrowly focused public health problem" (26).

We also did not anticipate that 19 of the 20 cases we studied would successfully enact smoke-free policies. This finding could be interpreted to mean that policy to create smoke-free outdoor areas is simple to enact; alternatively, we would argue that the projects in this study correctly assessed their local political climates and tailored their approaches accordingly. They were sufficiently sophisticated to propose voluntary or administrative policy change when they anticipated resistance to outdoor smoke-free policies, recognizing the need to postpone plans for adopting more stringent legislative policy.

One limitation of this study is that the findings are not necessarily generalizable, in part because of the unique support the local projects in this study received from the state tobacco control program. CTCP guarantees minimum funding to local health departments, encourages local policy change to reduce exposure to secondhand smoke, and hosts frequent training sessions for staff on community organizing and assessment, factors that give these projects certain advantages. Another limitation is that the number of projects for this comparative analysis was small. However, these 20 projects covered more than 200 communities in California, so the effect of their enacted policies is potentially large.

Smoke-free outdoor policies are a relevant new area in tobacco control efforts and are consistent with CDC's emphasis on eliminating tobacco smoke from public areas (8). To our knowledge, this study is the first to investigate the internal strategies that advance local smoke-free outdoor policies. More research on this topic is needed to determine whether these strategies are equally effective when used by nongovernmental agencies that are funded for a limited time and to isolate the factors that explain the diffusion of new outdoor smoke-free policies across jurisdictions. A careful examination of the effect of outdoor policies on protection from secondhand smoke, reduction of negative modeling for children, and reduction of litter and toxins from cigarette butts would add public health legitimacy to this policy approach.

## Figures and Tables

**Table:** Projects Described in the Final Evaluation Reports (FERs) Addressing Outdoor Smoking in Recreational Spaces

**County or City**	Outcomes	Type of Policy (Policy Body)
Del Norte County	Progressed from 1 smoke-free day at the county fair to all 4 days smoke-free except for designated areas during program periods.	Administrative (fair board)
El Dorado County	The county fair and local ski resort became smoke-free with designated smoking areas; 6 additional parks and 2 city pools also became smoke-free.	Administrative (fair board), voluntary (business), administrative (parks and recreation departments)
Humboldt County	Two policies enacted: 1) a no-smoking policy at the fairgrounds (except for designated smoking area) and 2) a city policy in Blue Lake covering outdoor spaces.	Administrative (fair board), legislative (city council)
Lake County	Reported that 19 outdoor community events had adopted and enacted smoke-free policies at the end of program period.	Voluntary (event coordinators)
Long Beach	Enacted a smoke-free beach ordinance.	Legislative (city council)
Los Angeles County	Smoke-free outdoor policies passed in 7 cities (also reported: smoke-free parks in 4 cities, smoke-free beaches in 5, other smoke-free policies in 5 more).	Legislative (city councils)
Marin County	The county board of supervisors adopted a comprehensive smoke-free policy that has been a model for several other communities.	Legislative (county board of supervisors)
Monterey County	Six events and venues adopted smoke-free policy.	Voluntary (event coordinators)
Nevada County	Six parks in 2 cities became smoke-free. Truckee City Council passed an ordinance for 3 parks and a skate park; Nevada City passed ordinances for 3 parks and 2 skate parks.	Legislative (city councils)
Orange County	Seven cities passed smoke-free park/beach ordinances covering more than 100 venues.	Legislative (city councils)
Pasadena	Four agencies adopted a voluntary smoke-free policy at 7 events.	Voluntary (event coordinators)
Riverside County	A total of 32 parks are covered by the no-smoking ordinances adopted by the 2 cities (Corona and Moreno Valley).	Legislative (city councils)
Sacramento County	Nine policies were adopted by 7 organizations (6 smoke-free events and 3 sponsorship policies).	Voluntary (event coordinators)
San Diego County^a^	Ten cities adopted smoke-free policies; 7 community events became smoke-free.	Legislative (city council), voluntary (event coordinators)
	One college adopted a smoke-free policy; 3 campuses created designated smoking areas.	Administrative (college board of trustees)
San Luis Obispo County	Two cities (Morro Bay and Pismo Beach) passed no-smoking policies covering their beaches and pier.	Legislative (city councils)
San Mateo County^a^	One city (Pacifica) passed a smoke-free ordinance for its beaches and pier.	Legislative (city council)
	Three local events became smoke-free.	Voluntary (event coordinators)
Santa Cruz County^a^	Three policies were adopted, covering 3 beaches and 2 city parks in Santa Cruz and Capitola and all county parks (Santa Cruz County).	Legislative (city councils), legislative (county board of supervisors)
	Smoke-free pride parades and festivals became smoke-free.	Voluntary (event coordinators)
Solano County	Board of supervisors approved a preliminary plan. No official policy enacted.	None
Stanislaus County	Passage of smoke-free baseball park complex in the city of Ceres.	Legislative (city council)
Yuba County	Thirteen events have become smoke-free (some have designated smoking areas).	Voluntary (event coordinators)

a Two final evaluation reports submitted.

## References

[1] Glantz SA, Parmley WW (1995). Passive smoking and heart disease: mechanisms and risk. JAMA.

[2] He J, Vupputuri S, Allen K, Prerost MR, Hughes J, Whelton PK (1999). Passive smoking and the risk of coronary heart disease: a meta analysis of epidemiological studies. N Engl J Med.

[3] Klepeis NE, Ott WR, Switzer P (2007). Real-time measurement of outdoor tobacco smoke particles. J Air Waste Manag Assoc.

[4] Novotny TE, Zhao F (1999). Consumption and production waste: another externality of tobacco use. Tob Control.

[5] Alesci NL, Forster JL, Blaine T (2003). Smoking visibility, perceived acceptability, and frequency in various locations among youth and adults. Prev Med.

[6] Gilpin EA, Lee L, Pierce JP, Tang H, Lloyd J (2004). Support for protection from secondhand smoke: California 2002. Tob Control.

[7] Klein EG, Forster JL, McFadden B, Outley CW (2007). Minnesota tobacco smoke-free policies: attitudes of the general public and park officials. Nicotine Tob Res.

[8] Centers for Disease Control and Prevention. Best practices for comprehensive tobacco control.

[9] Francis JA, Abramsohn EM, Park HY (2010). Policy-driven tobacco control. Tob Control.

[10] Kingdon J (1984). Agendas, alternatives, and public policies.

[11] Shipan CR, Volden C (2006). Bottom-up federalism: the diffusion of anti-smoking policies from US cities to states. Am J Poli Sci.

[12] (2004). Town-level characteristics and smoking policy adoption in Massachusetts: are local restaurant smoking regulations fostering disparities in health protection?. Am J Public Health.

[13] Feiock RC, West JP (1993). Testing competing explanations for policy adoption: municipal solid waste recycling programs. Pol Res Q.

[14] Bartosch WJ, Pope GC (1999). The economic effect of smoke-free restaurant policies on restaurant businesses in Massachusetts. J Public Health Manag Pract.

[15] Mintrom M (1997). Policy entrepreneurs and the diffusion of innovation. Am J Pol Sci.

[16] Skocpol T, Abend-Wein M, Howard C, Lehman SG (1993). Women's associations and the enactment of mothers' pensions in the United States. Am Pol Sci Rev.

[17] Samuels B, Glantz SA (1991). The politics of local tobacco control. JAMA.

[18] Roeseler A, Burns D (2010). The quarter that changed the world. Tob Control.

[19] Ulin PR, Robinson ET, Tolley EE (2005). Qualitative methods in public health: a field guide for applied research.

[20] Corbin J, Strauss A (2008). Basics of qualitative research.

[21] Glaser BG, Strauss AL (1967). The discovery of grounded theory: strategies for qualitative theory.

[22] Denzin NK (2003). Performance ethnography: critical pedagogy and the politics of culture.

[23] Patton MQ (2006). Qualitative research evaluation methods.

[24] Satterlund TD, Cassady D, Treiber J, Lemp C (2011). Barriers to adopting and implementing local-level tobacco control strategies. J Community Health.

[25] Browne WP (1985). Variations in the behavior and style of state lobbyists and interest groups. J Politics.

[26] Blaine TM, Forster JL, Hennrickus D, O'Neil S, Wolfson M, Pham H (1997). Creating tobacco control policy at the local level: implementation of a direct action organizing approach. Health Educ Behav.

